# Investigating the Synthesis and Characterization of a Novel “Green” H_2_O_2_-Assisted, Water-Soluble Chitosan/Polyvinyl Alcohol Nanofiber for Environmental End Uses

**DOI:** 10.3390/nano8060395

**Published:** 2018-06-01

**Authors:** Md. Nahid Pervez, George K. Stylios

**Affiliations:** Research Institute for Flexible Materials, School of Textiles and Design, Heriot-Watt University, Galashiels TD1 3HF, UK; nahid.tex92@gmail.com

**Keywords:** water soluble chitosan, green nanofiber, ecofriendly solvent, Hesperetin, Reactive black 5

## Abstract

The present work highlights the formation of a novel green nanofiber based on H_2_O_2_-assisted water-soluble chitosan/polyvinyl alcohol (W_S_CHT/PVA) by using water as an ecofriendly solvent and genipin used as a nontoxic cross-linker. The 20/80 blend ratio was found to have the most optimum uniform fiber morphology. W_S_CHT retained the same structure as W_IS_CHT. The prepared nanofibers were characterized by Scanning electron microscopy (SEM), Fourier transform spectroscopy (FTIR), Thermo gravimetric analysis (TGA), Differential scanning calorimeter (DSC), X-ray diffraction (XRD), Water Contact Angle (WCA) and Ultraviolet-visible spectroscopy (UV-vis). During electrospinning, the crystalline microstructure of the W_S_CHT/PVA underwent better solidification and after cross-linking there was an increase in the melting temperature of the fiber. Swelling ratio studies revealed noticeable increase in hydrophilicity with increase of W_S_CHT, which was further demonstrated by the decrease of contact angle from 64.74° to 14.68°. W_S_CHT/PVA nanofiber mats exhibit excellent UV blocking protection with less than 5% transmittance value and also showed improved in vitro drug release properties with stable release for longer duration (cross-linked fibers) and burst release for shorter duration (uncross linked) fibers. Finally our experimental data demonstrates excellent adsorption ability of Colour Index (C.I.) reactive black 5 (RB5) due to protonated amino groups.

## 1. Introduction

Nanotechnology is exploring an extensive range of fascinating materials that have outstanding chemical and physical properties. These materials include quantum dots or nanoparticles that are zero dimensional, nanowires which are one-dimensional, two-dimensional nanosheets, nanorods, and nanotubes [1]. In the category of nanomaterials, there is also a group known as nanofibers that differ from other nanomaterials because of their unique properties leading to different end uses. One property is that they can be porous while having a high surface area to volume ratio at the same time. This makes nanofibers suitable for various sophisticated applications including filters, textiles, tissue engineering, wound healing, soft sensors, drug delivery, catalysis, optoelectronics, and composites [2,3,4].

Considering various strategies of synthesizing nanofibers, a suitable and preferred technique is electrospinning [5,6,7]. The technique is widely utilized because of the ease of processing, the effective control of fiber diameter, low cost of processing, effectiveness, and reproducibility. Generally, the set-up of electrospinning comprises of a syringe that has a nozzle, a source of electric field, a grounded target or counter electrode and a metering pump. The process of electrospinning utilizes the electrostatic principles whereby the forces of electrostatic repulsion within a high electrical field synthesize nanofibers. Electrospun nanofiber physical properties depend on various parameters such as, environmental factors (e.g., humidity and processing temperature), solution properties (e.g., surface tension, viscosity, and conductivity), and process parameters (e.g., flow rate, collection distance and electrical potential) [8]. Several variations of conventional electrospinning exist. They include emulsion electrospinning, multineedle, suspension electrospinning, coaxial electrospinning, needleless, reactive electrospinning, green electrospinning, etc. [9] The materials generated from the green electrospinning technique are at a high demand since they provide an alternative means of protecting surfaces from a range of microorganisms. This protective property comes as a result of using water-based materials and solvents, which are cheap, environmentally friendly, and nontoxic. This is the focus of this research.

Recently, there is a great interest by academe and industry to manufacture green polymers that are derived from existing natural resources. The efforts are geared toward replacing petroleum-based polymers with natural biopolymers that are sustainable, environmentally friendly, biodegradable, and renewable [10,11]. Chitosan (CHT) is a natural polymer for producing bionanocomposites that have received great preference in medical and pharmaceutical uses because of its mechanical, chemical, and physical characteristics [12] Along with its biodegradability, nontoxicity, biocompatibility, and the ease it can be combined with several other chemicals lends itself to be investigated further for improving structure-use relationships [13]. For instance, chitosan, whose structure is sponge-like, was integrated with a growth factor derived from platelets then utilized as a bone regeneration osteoconductive material [14]. Also, porous chitosan containing calcium phosphate was used like a scaffold for delivery of antibiotic drugs [15]. Furthermore, chitosan could combine toxic and heavy metal ions like CrO_4_^2−^, Zn^2+^, Mg^2+^, and Cu^2+^ from aqueous solutions, because of the amine groups on its backbone [16,17]. Extensive research has been carried out on biopolymers and how they can assist in the synthesis of some tissue engineering scaffolds. This is due to the fact that biopolymers consist of proxy structures that are similar to that of glycosaminoglycan, which is an essential component found in the native extracellular matrix to facilitate cell proliferation and attachment as well as improving tissue and cellular biocompatibility, particularly those synthesized through electrospinning [18,19].

Despite, a lot of research being carried out to find more applications of chitosan nanofibers, its production remains challenging. Chitosan nanofibers exhibit a high affinity to hydrogen bonding and they are also highly crystalline. As a result of these properties, they are less soluble to common organic solvents and water [20]. For the latter, due to difficulties in processing chitosan, additives that facilitate formation of fibers like polyethylene oxide and polyvinyl alcohol (PVA) are added to facilitate the process of electrospinning [21]. Among these additives, polyvinyl alcohol is regarded as the best additive owing to its water permeability, biocompatibility, nontoxicity, and biodegradability. Furthermore, hydroxyl groups present in polyvinyl alcohol are extensively used in biomedical and biochemical applications. Polyvinyl alcohol has high hydrophilic properties and the potential of forming good fiber, which has resulted in its commercialization since the 1950s. Polyvinyl alcohol is a distinctive category of fiber because it is easily coagulated, spun, cross-linked, and oriented [22]. Previously, great efforts have been put in place to facilitate fabrication of a high surface area nanofiber blended with CHT/PVA using the electrospinning technique [23,24,25]. However, both expensive and toxic chemicals like dichloromethane, trifluoroacetic acid (TFA), acetic acid, and chloroform may be used, but their residues negatively impact the health of the user and the environment in general, thus they cannot be categorized as green nanofibers. Therefore, functional fibers of that nature are rarely used in a variety of anticipated applications in areas with strict adherence to environmental safety and nontoxicity principles.

Having been inspired by the aforementioned considerations, we report a novel synthetic technique to fabricate H_2_O_2_-assisted water-soluble chitosan/polyvinyl alcohol (W_S_CHT/PVA) blended ‘green’ nanofiber, which is the first attempt in the literature. The chemical synthesis and the optimum processing parameters of the nanofibers are investigated and discussed. The nanofiber blends were characterized by SEM, FT-IR, TGA, DSC and XRD, and multifunctional properties like, UV protection, in vitro drug release of hesperetin and catalytic studies on Reactive Black 5 were investigated.

## 2. Materials and Methods

### 2.1. Materials

High molecular weight chitosan (degree of deacetylation 65%), Genipin and C.I reactive black 5 (M_w_: 991.82 g mol^−1^) were purchased from Sigma Aldrich, Dorset, UK. Poly (vinyl alcohol), (PVA, M_w_: 115,000) was purchased from BDH Limited Poole England and Hesperetin was purchased from Alfa Aesar, Lancashire, UK. The following chemicals were all obtained and used as reagents: sodium hydroxide, hydrogen peroxide, hydrochloric acid and ethanol. All chemicals were used without further purification.

### 2.2. Synthesis of Water Soluble Chitosan (W_S_CHT)

The following synthesis protocol was based on a previously reported method [26] with a few adjustments. For the sake of clarity, chitosan was completely dissolved in 1% (*v*/*v*) aqueous hydrochloric acid to a concentration of 1% (*w*/*v*), then 30% (*v*/*v*) H_2_O_2_ aqueous solution was added into solution and then kept in a thermostatic water bath at 65 °C for 40 min. The final solution was then filtered by utilizing Whatman filter paper to expel polluting influences and for promoting decontamination of final product was acquired by means of dialysis using a Slide-a-Lyzer dialysis cassette (Pierce, MWCO 2000) (molecular weight cut-off) in deionized (DI) water for 12 h at room temperature. At that point, the pH of the solution was brought to 7 by adding a few drops of 5 M NaOH solution and precipitation was obtained by adding ethanol. This step was followed by another rinsing with hot water a couple of times to ensure complete removal of NaOH. Then collected solid after drying the precipitate in vacuum, was washed with ice cold water in order to remove the excess traces of acid and gently casted onto polystyrene dishes and dried at 25 °C to obtain pure water soluble chitosan (W_S_CHT).

### 2.3. Solution Preparation for Electrospinning

**Scheme 1:**
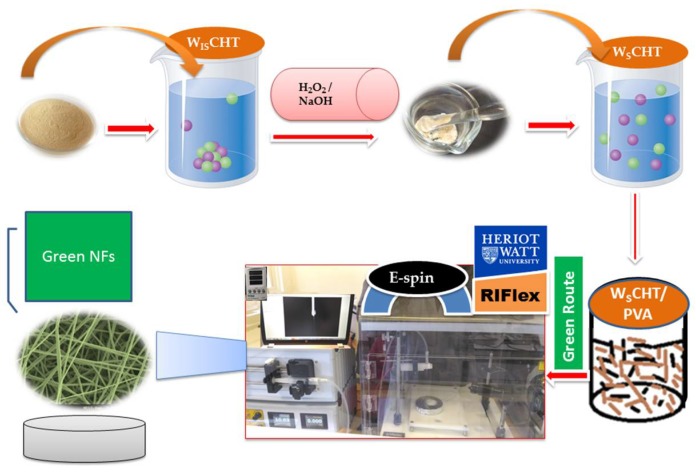
Schematic of synthesis of “green” W_S_CHT/PVA nanofiber.

Firstly, W_S_CHT solution (0.7% concentration) was prepared by dissolving W_S_CHT powder in water (milli-Q grade) with gentle magnetic stirring for 56 h at room temperature to form a homogeneous solution PVA solutions (9.3% concentrations) were prepared by dissolving in double-distilled water under magnetic stirring for 4 h at 75–80 °C, and then cooled to room temperature. W_S_CHT/PVA blend solutions were then prepared by mixing the two solutions at 10/90, 15/85, 20/80, 25/75 and 30/70 W_S_CHT/PVA mass ratios. All electrospinning experiments were carried out on an electrospinning instrument (Research Institute for Flexible Materials, Heriot-Watt University, Edinburgh, UK) under Scheme 1. The prepared blend solution was drawn into a 5 mL disposable plastic syringe equipped with a metallic needle (inner diameter gauge of 22). The flow rate was monitored by a syringe pump. A high voltage power supply was applied to the solution in order to generate electrostatic field between the grounded collector (copper collector plate measuring 15 cm × 15 cm) and the syringe needle tip. Aluminium foil was placed over the grounded collector plate to collect the electrospun nanofibers. Exploratory investigations established the optimum process parameters which were kept throughout this study; voltage 16 kV, flow rate 0.7 mL h^−1^ and distance 8 cm. Finally, the collected nanofibers were dried at 25 °C under vacuum for 24 h before they were detached to remove the residual solvent. All experiments were conducted at room temperature with a relative humidity of 35 ± 5%.

### 2.4. Green Cross-Linking

For green electrospinning, the prepared nanofiber was placed into a PBS (pH 7.4) solution containing (0.5% *w*/*v*) concentration of genipin (GP) at 37 °C for 24 h. The cross linking process was completed by washing the nanofiber in PBS solution and dried over two days in a vacuum oven to remove any potential residual solvents.

### 2.5. Characterization

The viscosity of the solutions was measured using a viscometer (DV-II, Brookfield Co., Stoughton, MA, USA). The surface tension of solutions was tested on a surface tension meter (KRÜSS GmbH, Hamburg, Germany). The conductivity of the solutions was measured on a conductivity meter (HANNA HI8733, Leighton Buzzard, UK). All measurements were carried out at room temperature. The surface morphologies of the electrospun nanofibers were examined with a scanning electron microscopy (SEM; Hitachi, S-4300, Tokyo, Japan) at accelerating voltage of 3–5 kV. The diameters of nanofibers were measured by using an image analyzer (Image J, National Institutes of Health, Bethesda, MD, USA). Fourier transform spectroscopy (FTIR) was obtained by a Thermo Nicolet Avatar 370 DTGS spectrometer using KBr disc containing ground sample particles. Differential scanning calorimeter (DSC) was measured on a Mettler DSC 12E at 10 °C min^−1^ heating rate. Thermogravimetric analysis (TGA) was performed on a Mettler Toledo TGA in N_2_ atmosphere with a heating rate of 10 °C min^−1^ at a temperature range between 30 °C to 800 °C. X-ray diffraction (XRD) patterns were collected on a Bruker D8 Advance X-ray diffractometer with Cu Kalpha1 radiation.

### 2.6. Swelling Ratio

The swelling ratio was investigated by immersion in phosphate buffered saline (PBS) (pH 7.4) at 20 °C with gentle shaking [27]. Twenty-four hours later, the weight of the swollen scaffold was measured and the swelling ratio was figured with the following equation:

(1) $$E_{\mathrm{sw}}\;=\;\frac{W_{\mathrm{sw}}\;-{\;W}_{o}}{W_{o}}\;\times 100\%$$

where *E*_sw_ is the swelling ratio of the nanofiber, *W*_o_ is the initial dried weight of the nanofiber and *W*_sw_ is the weight of the swollen nanofiber.

### 2.7. Water Contact Angle

Water contact angles of nanofibers were measured by a contact angle meter (Dyne Testing, UK) at ambient conditions by dropping 5 µL of deionized water on the surface of samples. The contact angles were measured using a sessile drop method, and average of five measurements was reported.

### 2.8. UV Protection

The UV-protection properties of the nanofiber mats were evaluated according to Australian/New Zealand Standard AS/NZS 4399 (1996). The UV transmission spectra were recorded three times in different directions at wavelengths of 200–400 nm using a UV-vis spectrophotometer (Perkin-Elmer Lambda 950, PerkinElmer, Inc., Shelton, CT, USA).

### 2.9. In Vitro Drug Release Study

The in vitro drug release study for hesperetin from W_S_CHT/PVA nanofiber (sample c-IS) and cross-linked sample (CR-IS) was incubated in 10 mL of phosphate buffer solution (PBS) (pH = 7.4) at 37 °C. At various time points, 2 mL of the release medium was removed and followed by diluting to 10 mL using fresh buffer solution. The amount of released hesperetin was determined using the UV-vis spectrophotometer at the wavelength of 288 nm [28].

### 2.10. Dye Removal Efficiency

CI Reactive Black (RB5) dye is a diazoacidic reactive dye, which consists of two sulfonates and two sulfato-ethylsulfon groups, typically used in textile industry [29] was selected for adsorption studies. The pristine W_IS_CHT, W_S_CHT, W_S_CHT/PVA nanofiber (sample c-IS) and cross-linked of sample (CR-IS) nanofiber mat samples (0.02 g) were individually placed in a batch containing 250 mL solution of RB5 with a concentration of 30 mg L^−1^ to measure the dye removal efficiency (%). The pH of each solution was adjusted to the desired value using HCl or NaOH solution. The amounts of dye removal from solutions were determined as a function of time according to the following equation:

(2) $$DR\;(\%)=\frac{A_{o}\;-\;A}{A_{o}}\;\times 100$$

where *DR* is dye removal, *A*_o_ and *A* are dye concentration at t = 0 and t, respectively.

## 3. Results and Discussion

### 3.1. Synthesis of Water Soluble Chitosan (W_S_CHT)

Since CHT has a high molecular weight and low solubility in most solvents, which limits its applications in different fields [30]. By the degradation process CHT can be converted into the low-molecular weight CHT, which exhibits good water solubility. Thus, in this study we used high molecular weight CHT; denoted as water insoluble CHT(W_IS_CHT) starting material to get low molecular weight CHT; termed as water soluble CHT(W_S_CHT) through catalytic oxidation with hydrogen peroxide which is in demand because of economic and environmental benefits.

**Figure 1:**
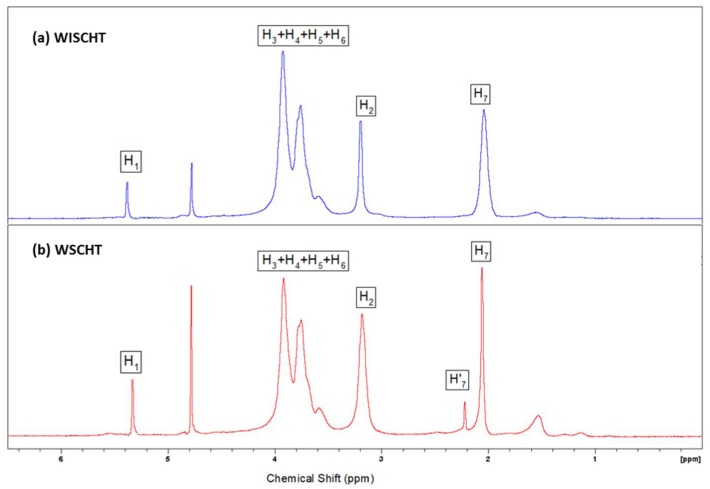
Proton nuclear magnetic resonance (^1^H NMR) spectra of (**a**) W_IS_CHT and (**b**) W_S_CHT.

Endeavoring to better comprehend the idea of the modified CHT, we performed proton (^1^H) Nuclear magnetic resonance (NMR) spectroscopy of samples. It has been generally acknowledged as a basic instrument that provides data on the chemical environment and structure of materials that are not noticeable by other nonruinous high-determination spectral methods [31]. The ^1^H NMR spectrum of W_IS_CHT and W_S_CHT is shown in Figure 1a,b. In the case of W_S_CHT, it exhibits an absorption peak at 2.091 ppm (H-7) corresponding to the ring methane protons because of the *N*-acetyl glucosamine units having survived the saponification chitin, and a singlet at 3.20 ppm (H2) and four absorption peaks at 3.67–4.02 ppm (H-3, H-4, H-5, and H-6), and also a small signal appeared at 5.34 ppm (H1). Compared with the ^1^H NMR spectrum of W_IS_CHT, the shift of each ^1^H of W_S_CHT is corresponding to the shift of CHT, so the significant changes in characteristic shifts of W_S_CHT do not take place when compared to W_IS_CHT in the spectra. In other words, the structure of W_S_CHT remains the same as that of the W_IS_CHT which supports the previous findings in literature [26]. The degree of deacetylation (DD %) is defined as the molar fraction of GlcN in the copolymers (CHT) composed of GlcNAc and GlcN and the DD value is one of the most important factors in assessing end uses in medical, nutritional, sewage treatment, and biotechnology fields [32]. NMR spectroscopy is one of the most accurate methods for determining the degree of deacetylation, and it has been chosen as a standard method by the American Standard Test Method [33]. The degree of deacetylation of W_IS_CHT and W_S_CHT obtained 65% and 88%, respectively, which is standard.

### 3.2. Solution Properties and Morphological Behaviour

It is well-documented that the major physical properties of a solution (surface tension γ, conductivity σ, and viscosity) played an important role in the process of electrospinning, thus affecting the electrospun’s fiber final characteristics, as illustrated in Table 1 [34,35]. The viscosity, η of any polymer solution, is essential since it determines the nanofibers’ morphology. Solutions with low viscosity do not generate uniform and continuous fibers, while those with high viscosity produce jet ejections of nonuniform polymer. As a matter of fact, viscosity that is dependent on interactions between polymer-solvents is what determines the possibility of a polymer solution to be electrospun [36]. The results illustrate that the viscosity of water-soluble chitosan and poly vinyl alcohol blended solution decreases with increasing of water-soluble chitosan content. The major decrease in viscosity is as a result of removal of acetyl groups from the W_S_CHT backbone and the reduction of polymer chains in order to form shorter chains by hydrolysis [37]. Normally, this affects the diameter and morphology of nanofiber mats: a decrease in the viscosity of a solution decreases the nanofiber diameter and bead formation in mats. As seen, the conductivity of a blend increases when W_S_CHT content increases. When content increases, there is more protonation of –NH_2_ groupings of W_S_CHT, which is in aqueous solution. As W_S_CHT content increases the surface tension of the blend increases as well. While various solvents have low surface tension, a higher surface tension is experienced in water, making the process of electrospinning using water more difficult since the jet is susceptible to breaking, forming droplets or beaded fibers. This study demonstrates that, when W_S_CHT is increased in water solvent, it increases the solution’s surface tension because of the inherent nature of water with a high surface tension.

**Figure 2:**
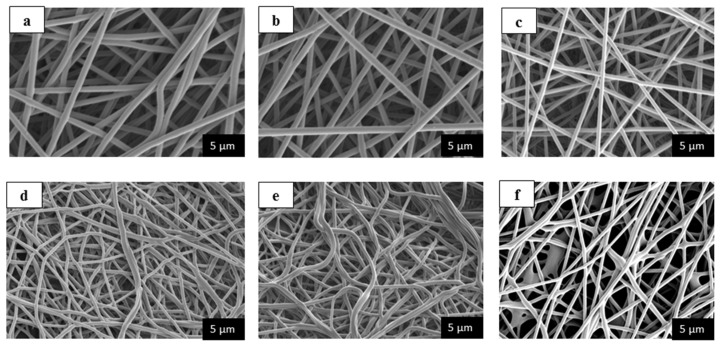
Scanning electron microscopy (SEM) images of W_S_CHT/PVA nanofibers; (**a**) 10:90; (**b**) 15:85; (**c**) 20:80; (**d**) 25:75; (**e**) 30:70 and (**f**) Crosslinking of CR (**c**) 20:80.

**Figure 3:**
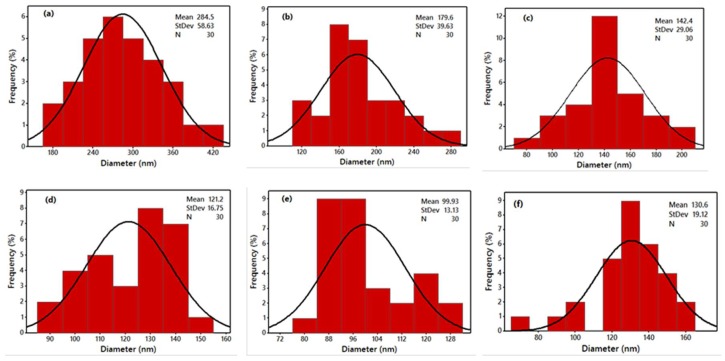
Diameter distribution histograms of W_S_CHT/PVA nanofibers; (**a**) 10:90; (**b**) 15:85; (**c**) 20:80; (**d**) 25:75; (**e**) 30:70; and (**f**) Crosslinking of CR (**c**) 20:80.

Figure 2a–e demonstrates SEM images of various nanofiber mats with diverse W_S_CHT-PVA blended ratios. When W_S_CHT quantity was increased, there was a decrease in the diameter of nanofibers to 99.93 nm from the original 284.5 nm shown in Table 2. Figure 3a–e demonstrates the distribution of diameters of nanofiber mats. This outcome is similar to other studies carried previously on nanofiber membranes blended PVA/CHT [38]; there was a decrease in diameter as contents of CHT increased in the blend, and as a result the number of beads created in the composite increased. CHT is basically an ionic polyelectrolyte that causes a high-charge density on the ejected jet’s surface during electrospinning. As the charges on the jet increase, extensive forces of elongation are forced on the jet under electrical field. It is well known that the fibers’ overall tension is dependent on the self-repulsion of extra charges that are on the jet. Thus, it increases in charge density and decreases the diameter. These results reveal that higher W_S_CHT content in the blend ratios lowers viscosity. Lower viscosities hinder polymer chain entanglement as well as reducing the viscoelastic forces. Therefore, an electrified-solution jet does not solve the higher force of Coulombic stretching during the process of electrospinning and these result to beads, fiber breaks, as well as instability encountered during the process [39]. The samples (d) and (e) fabricate fibers that have spindle-like structures. Furthermore, as the ratio of PVA is decreased, mere electrospraying is performed with the formation of nonsuitable nanofibers. This occurrence results from the increase in the surface tension of the solution, which is affected by the amount of voltage and charge density applied. Finally, sample (c) illustrates fabrication of more aligned nanofibers as compared to the other samples. Based on our results, characterization and cross-linking were applied only to sample (c) and noted as CR (c).

### 3.3. Green Cross-Linking

Next, our interest is turned on the cross-linking behavior of the produced nanofiber. Polymer cross-linking is being used to improve the mechanical and physical properties of polymers. The technique also enhances polymers’ resistance to water. Cross-linking is done by utilizing multifunctional agents that are able to react chemically with functional groups that are present on either molecules or proteins. Cross-linking improves the water stability of most starches and it also reduces swelling. Furthermore, films that are cross-linked exhibit higher resistance and thermal stability to degradation compared to other films that are not cross-linked [40]. Formerly, glutaraldehyde (GTA) has been used as a cross-linker during the process of cross-linking CHT nanofibers. However, the use of GTA resulted in the production of nanofiber CTS membranes that have increased brittleness, color change, as well as reduced tensile strength. Furthermore, increased cytotoxicity of GTA poses another issue of concern to the environment and end users [41]. 

Alternatively, research has established that genipin (GP), which is a chemical compound extracted from the fruits of the *Gardenia jasminoides* Ellis plant can be utilized as the best green cross-linker for the process of cross-linking both polysaccharides and proteins biopolymers that contain amine groups as primary residues [42]. As mentioned earlier in this discussion, the focus is geared toward production of ecofriendly nanofibers. For instance, genipin, which is used as cross-linker, results in green nanofibers and is why this agent is chosen in this study. Figure 2f portrays the SEM images of nanofibers of sample (c) that have undergone cross-linking. After the process of cross-linking, the electrospun nanofiber changed its colour to yellow. This is related with the oxygen radical-induced polymerization of genipin that is induced with oxygen radicals and the subsequent reaction with amino groups contained in W_S_CHT [43]. Although other fibers shrink from their normal dimensions as a result of decreasing size between inter-fiber pores, the process of cross-linking W_S_CHT/PVA retains the morphology of nanofibers with reduced diameter Figure 3f.

### 3.4. Fourier Transform Infrared Spectroscopy (FT-IR)

**Figure 4:**
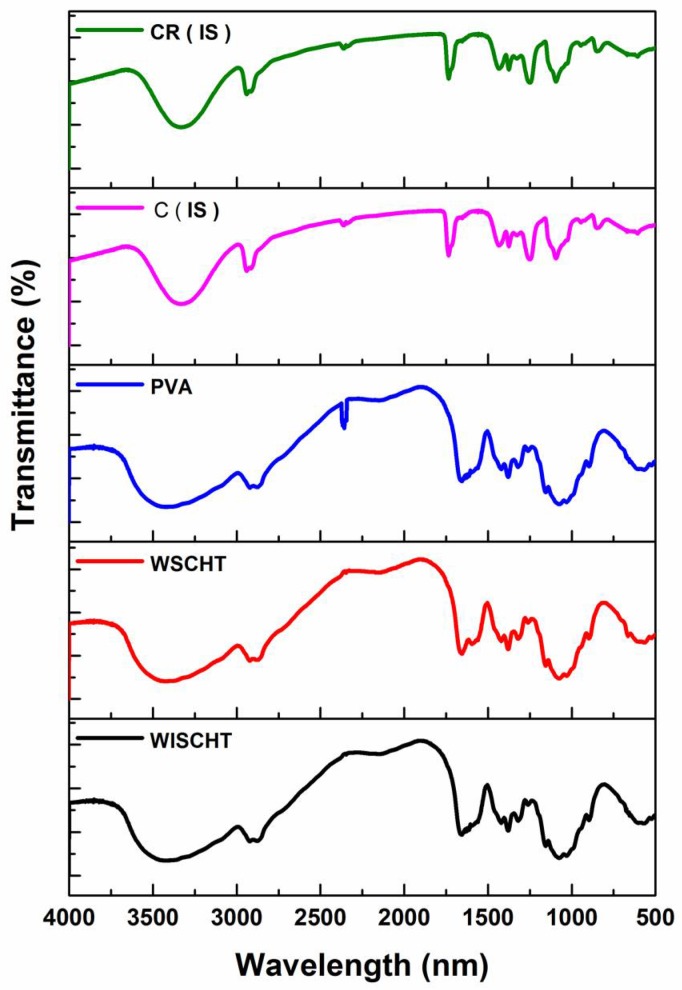
Fourier transform spectroscopy (FT-IR) spectra of prepared samples.

FT-IR spectroscopy is a sensitive and affective technique of detecting the chemical bonds of materials. Figure 4 illustrates the various FT-IR spectra of W_IS_CHT, W_S_CHT, PVA, C (IS) and CR (IS). In W_IS_CHT, the peak band of approximately 3422 cm^−1^ is greatly attributed to N–H and O–H stretching vibration. The absorption peak of around 1599 cm^−1^ relates to the amino groups binding vibrations. Also, a band of carboxyl of around 1654 cm^−1^ is credited to the acetyl residual. The band range between 896 cm^−1^ to 1157 cm^−1^ are part of unique absorb peaks of b-1, 4 glycosides’ bonds found in water insoluble chitosan [44]. When a comparison is done between FT-IR spectrum of water insoluble CHT and that of a water soluble CHT, W_S_CHT produces a new peak of around 777.5 cm^−1^ and this is attributed to the decomposition of N=CHCOO^−^ group that results from N=C double bond [45]. This implies that a reaction between the –NH_2_ of CHT and –CHO of 2,5-anhydro-d-mannose end occurred. Most peaks that relate to acetate groups are revealed in the PVA FT-IR spectrum. Particularly, the broad band of 3200 to 3550 cm^−1^ is connected to O–H stretch from both intra-molecular and intermolecular bonds of hydrogen. Also, the vibration band recorded in ranges between 2840 cm^−1^ to 3000 cm^−1^ results from the C–H stretching from the original alkyl groups and the other peaks that ranges between 1680 cm^−1^ to 1730 cm^−1^ are as a result of C–O and C=O stretches that come from acetate groups that remain in the PVA [46].

Furthermore, the FT-IR spectrum of W_S_CHT-PVA nanofiber is an example of distinguished peaks of W_S_CHT apart from those that closely relate to ionization of the basic amino groups of water soluble chitosan. These peaks are at about 1408 cm^−1^ as well as between 1548–1560 cm^−1^. The peaks’ formation of 1552–1558 cm^−1^ results from symmetric decomposition of –NH^3+^ groups and peaks of about 1408 cm^−1^ is due to carboxylic acid. The peaks between 1700 and 1725 cm^−1^ characterize the dimers of carboxylic acid [47]. Also, another band at about 920 cm^−1^ is recorded because of the interaction between PVA and W_S_CHT. The observations above show the presence of excellent miscibility between CHT and PVA. This is possible due to the creation of inter-molecular hydrogen bonds between –NH and –OH groups in the PVA. Schiff base formation can explain the formation of samples of cross-linked nanofibers as demonstrated by bands at 1430 and 1634 cm^−1^ which are associated with NH_2_ and C=N groups, respectively [48]. During the cross-linking process, more covalent chemical bonds are formed in water soluble chitosan amino groups compared to PVA hydroxyl groups.

### 3.5. Thermogravimetric Analysis (TGA)

**Figure 5:**
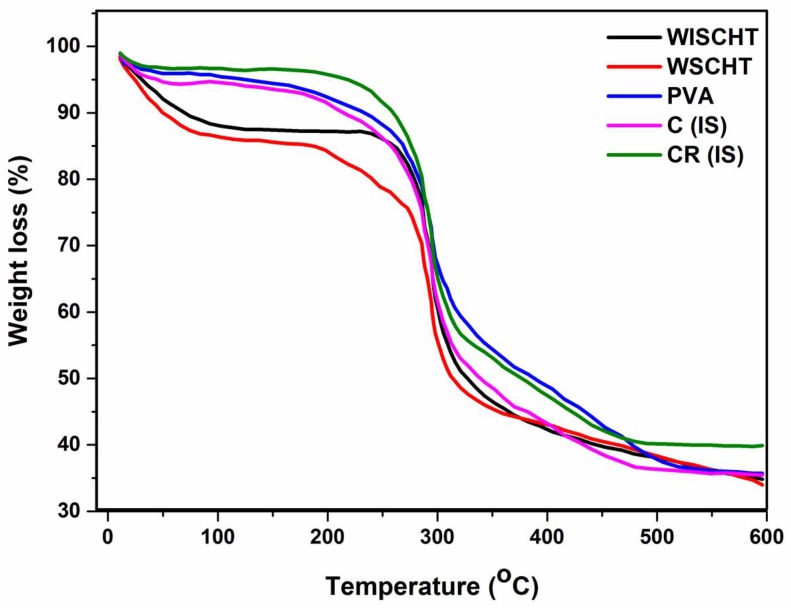
Thermogravimetric Analysis (TGA) of prepared samples.

The thermogravimetric analysis (TGA) is a widely used method to investigate the thermal decomposition behaviour of a polymer [49]. The TGA thermograms of W_IS_CHT, W_S_CHT, PVA, C(IS) and CR(IS), are shown in Figure 5. Figure 5 reveals that W_IS_CHT and W_S_CHT exhibit a two-step thermal degradation pattern. The first step occurs between 50 °C and 170 °C, which is attributed to loss of the adsorbed and bound water and the second step occurs between 185 °C to 480 °C with polymer degradation and a 55% plus polymer weight loss [50]. W_S_CHT shows less thermal stability than W_IS_CHT due to the differences of their molecular weight and structure. Prior to degradation, chitosan requires a lot of heat in order to break down complex hydrogen bonds that exist in the molecules. Also, degradation using hydrogen peroxide results in disintegration of both intermolecular and intramolecular interaction and this leads to partial disintegration of molecular structures [51]. The TGA curve of raw PVA shows three zones where mass loss is witnessed and this corresponds to the decomposition its components, which is in agreement with literature [52]. After the first step of water loss, decomposition occurs in the second step of degradation, which commences at 310 °C. At this temperature, 77% of the total mass is lost and this reflects PVA’s chain of decomposition. The subsequent step at 430 °C corresponds to PVA’s major chain degradation. A residue of about 4.5% of the starting mass is observed at 510 °C. The ideal sample of W_S_CHT/PVA nanofiber exhibited also a three-step degradation pattern. The first step occurs between 50 °C and 120 °C, due to water evaporation. The second and major mass loss is observed between 200 °C and 400 °C, here there is significant degradation of PVA and W_S_CHT. A smaller mass loss occurs between 450 °C and 550 °C. The cross-linked sample shows the same three degradation pattern with more thermal stability than the uncross-linked (ideal) sample, because of the cross-linking of networks along its macromolecular chain.

### 3.6. Differential Scanning Calorimetry (DSC)

**Figure 6:**
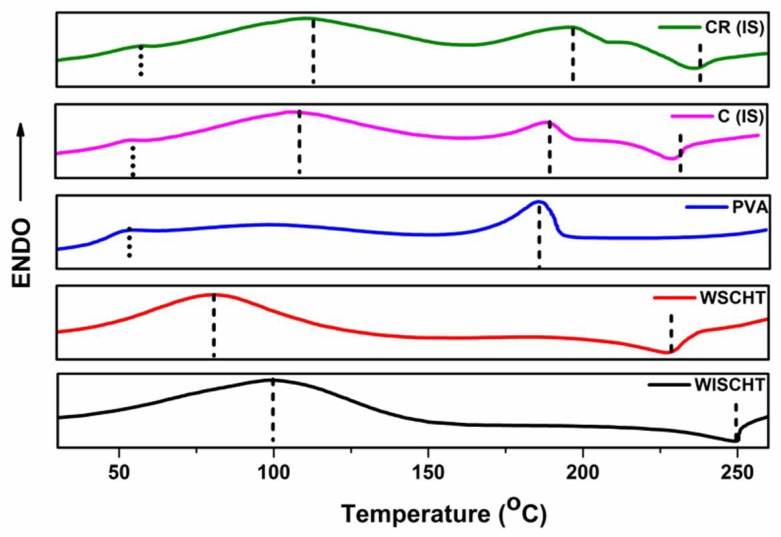
Differential Scanning Calorimetry (DSC)of prepared samples.

DSC is used to determine the crystallization and thermal parameters of our samples [53]. This technique can be utilized with composite precursors and composites to learn the thermodynamic processes like specific heat capacity and glass transitions, as well as kinetic events that include enthalpic relaxation and cure that is related to stress or physical aging of the polymer. The DSC thermograms of W_IS_CHT, W_S_CHT, IS, PVA and CR-IS at the heating rate of 10 °C min^−1^, to a temperature of 250 °C under nitrogen, have been investigated. Figure 6 illustrates the DSC curves produced from all samples. W_S_CHT and W_S_CHT produced an endothermic peak; water soluble chitosan exhibited a higher endothermic peak compared to water insoluble chitosan of 50 °C to 150 °C, owing to water vaporization within the polysaccharide backbone. Also, an additional exothermic event is recorded around 230 °C to 250 °C, due to decomposition resulting from the crystalline nature and the reduced weight of the material. In the case of PVA, an extensive endothermic peak is recorded at 54 °C. This is due to release of moisture of the water from the PVA powder. The start of the PVA melting peak is recorded at around 188 °C. Various reports claim that PVA glass transition peaks between 45 °C and 60 °C. Usually, DSC thermograms of water insoluble chitosan-PVA nanofibers, as represented by IS (ideal sample), portray three different shift peaks; at 180.6 °C, 118 °C and 230.2 °C. This shows that PVA and W_S_CHT retain their characteristic peaks. Small decreases as well as endothermic broadening melting temperature to 230.2 °C point out that there is a reduction in the degree of the polymer’s crystallinity [54]. It is evident that during electrospinning, the crystalline microstructure of the electrospun fiber underwent better solidification of the stretched chains. After cross-linking, there was an increase in the samples’ melting temperature. This is due to higher molecular orientation of the fiber aggregates. Therefore, the chemical cross-linking technique increases the melting temperature of the polymer.

### 3.7. X-ray Diffraction (XRD)

**Figure 7:**
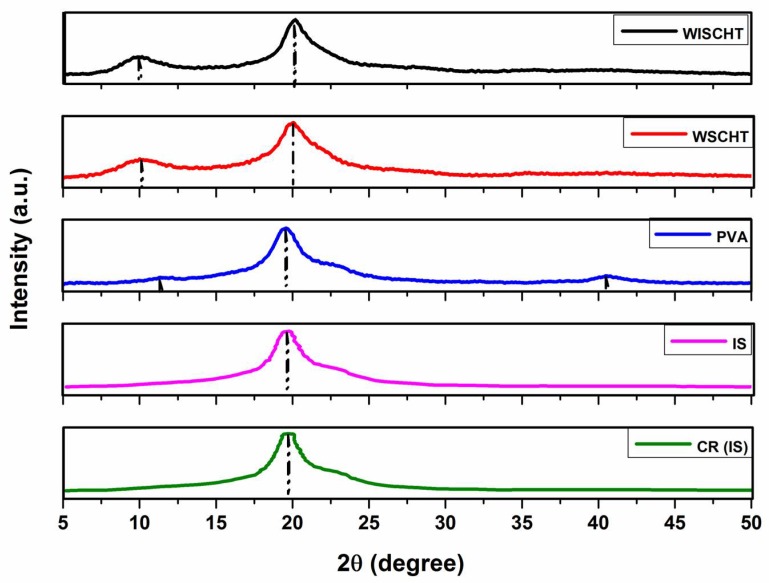
X-ray Diffraction (XRD) of prepared samples.

Supramolecular order of materials, also known as crystallinity, is investigated using an X-ray diffraction. Figure 7 shows X-ray diffraction patterns of W_S_CHT, W_IS_CHT, IS, PVA, and CR(IS). W_IS_CHT and W_S_CHT show two different peaks at 10–12 °C, as well as a sharp peak at 20–22 °C. In this case, W_S_CHT exhibits lower peak compared to W_IS_CHT. This is an indication that W_S_CHT, which is accelerated using hydrogen peroxide, contains a more crystalline structure as compared to W_IS_CHT [55]. The main X-ray diffraction peaks of PVA is at 19.50° and that of minor diffraction at 11.16 °C and 43.30 °C [56]. These peaks show that there are various crystal types in the PVA structure, resulting from high degree of crystallinity. Furthermore, contrary to other powders, the nanofiber sample that is blended with W_S_CHT-PVA demonstrated a peak at 19.6 °C. The respective peaks of W_S_CHT-PVA disappeared in the blended nanofiber sample owing it to the strong interaction between PVA and W_S_CHT. Although both structures of the cross-linked and un-cross-linked samples remained constant, there was a higher intensity and sharpness of the peaks of the cross-linked samples compared to those that were not cross-linked. This difference is due to high crystallinity experienced in those samples that are not cross-linked. The formation of uniform fibers increases when the crystallinity of the sample is higher, as is illustrated in Figure 2. The process of chemical cross-linking through PVA intermolecular interactions with water soluble chitosan are considerable with each of the blended solutions under investigation, in agreement with previous work [57].

### 3.8. Swelling Ratio (SR)

**Figure 8:**
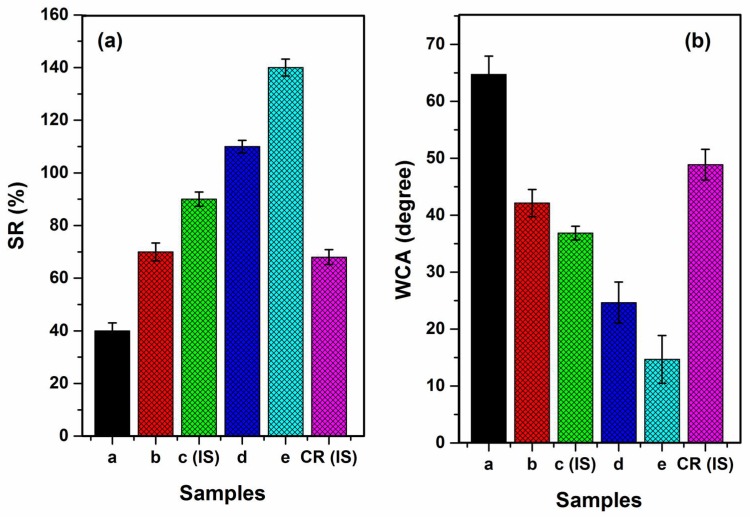
(**a**) Swelling ratio (SR%) and (**b**) water contact angle (WCA).

In order to examine the stability of nanofibers in terms of their structure, the swelling ratio is used. The swelling ratio of our samples is shown in Figure 8a. An increase in the swelling ratio to 145% from 40% prompted an increase in the ratios of W_S_CHT/PVA to 30/70 from 10/90. This is a clear indication that the hydrophilicity of nanofibers can be improved by the addition of water soluble chitosan. This comes as a result of secondary interactions that occur between PVA and W_S_CHT, causing polymer dissolution via hydrolysis. The process creates relatively short chains of the acid in that medium, thus enhancing hydrophilicity. Hideko et al. generated similar results [58]. When fragments of various polymers interact, a steric effect is created and this limits water absorption and causes the swelling rate to increase with a decrease in the amount of blended chitosan, and vice versa. In our investigation it is also evident that the swelling ratio associated with the (ideal) cross-linked samples (IS) is significantly lower compared to that of the uncross-linked samples. The same way that cross-linking reduces hydrophilicity, as well as the formation of intramolecular and intermolecular bonds between PVA macromolecular chains and water soluble chitosan, the cross-linked nanofibers have the same capability of reducing water absorption to a great extent and this contributes to maintaining stable fibrous structure in wet environmentals, in agreement with [27].

### 3.9. Water Contact Angle (WCA)

The study of water contact angle allows us to determine the hydrophobic or hydrophilic nature of any materials. Such descriptions can explain the lipase immobilization of the material. The angle of contact can be defined as the angle that lies between a plane tangent of a droplet of a liquid to that of a plane surface onto which the liquid settles. Surface wettability is dependent on the thermodynamic balance between interfaces of either a solid, vapour, or liquid. Therefore, the angle of contact θ represents a quantitative measure of the wetting process. When this point lies between 0° < θ < 90°, it implies that the liquid used wets the solid’s surface. When the point is between 90° < θ < 180°, the liquid used does not wet the surface of the solid [59]. Figure 8b demonstrates the various water contact angles obtained when droplets of water are placed on various nanofibers’ surfaces that have been blended with W_S_CHT/PVA. The water contact angle of various samples is reduced from 64.74° to 14.68° as the ratios of W_S_CHT/PVA increases from 10/90 to 30/70. This indicates that chitosan increased hydrophilicity of nanofibers (W_S_CHT/PVA). The reduction of the contact angle may be as a result of the Wenzel wetting model that happens on heterogeneous surface geometries [60]. Once the process of cross-linking is completed, there is an increase in the nanofiber’s contact angle to 48.88 CR (IS) from 36.86 (IS). From these results, it is evident that the process of cross-linking significantly affects the resistance of water of the W_S_CHT/PVA nanofiber.

### 3.10. UV Protection

**Figure 9:**
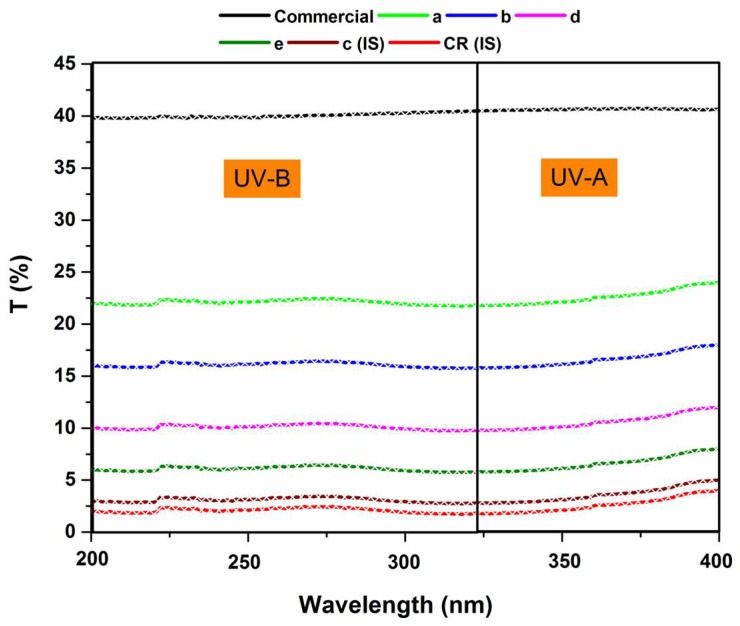
UV transmission spectra of commercial and prepared nanofiber samples.

The ultraviolet radiations can be categorized into UV-C (200–280 nm), UV-B (280–315 nm) and UV-A (315–400 nm). Of these radiations, only UV-B and UV-A reach the surface of the earth. UV-C is often absorbed before reaching the earth’s surface by ozone [61] in the stratosphere. Both UV-B and UV-A have positive and negative effects on the environment. For instance, they pose health issues such as skin cancer and immune system suppression. As a result, these health issues have prompted focus on functional fibers that have UV-protective characteristics [62]. In order to find out the ultra-violet blocking attributes of W_S_CHT/PVA nanofiber mats, the ultra-violet transmission curves of various samples were recorded at room temperature, as illustrated in Figure 9. It is known that 40% of both UV-B and UV-A radiation penetrates commercial samples. The transmittance values of nanofiber samples shows a dramatic decrease indicating that they have high ultra-violet blocking ability, due to superior volume surface to ratio. The results acquired from UV-blocking reveal that an increase in the ratio of water soluble chitosan also increases the UV-blocking efficiency properties. In fact, when ultraviolet transmittance is lower than 5%, the ultraviolet protection of any fabric material is considered as excellent, in agreement with Shahidi and Buzaytoun [63].

### 3.11. In Vitro Drug Release Study

Irrespective of CHT’s superiority as a great biomaterial, it does not fully dissolve in water, dissolving only in acidic solutions due to its strong crystallinity and deacetylation. This restricts its uses as a bioactive agent carrier for delivery of drugs, genes and peptides. Water soluble chitosan however, has been given special attention, since it dissolves easily in neutral aqueous solutions. This has a number of advantages, due to the ease with which it can be functionalised. Consequently, it can be used as a gene hydrophilic drug carrier with outstanding biodegradability, biocompatibility and nontoxicity [64].

**Figure 10:**
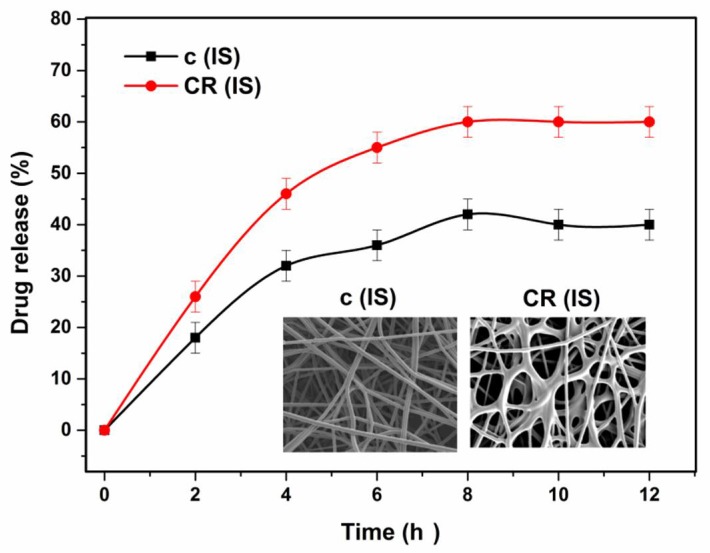
In vitro drug release assay of the prepared sample.

An examination of functionalised W_S_CHT/PVA nanofiber used for drug delivery was carried out in order to find out the vitro release characteristics as a drug delivery system. First, a study on drug release profile was carried out on the (ideal) sample and then on the cross-linked sample. The outcomes of the two release profiles are given in Figure 10. It is observed that the cross-linked nanofiber sample showed a stable release rate for a longer duration, while the uncross-linked nanofiber sample experienced a burst release once immersed in PBS. The burst release experienced in the uncross-linked nanofiber results from drug content accumulation on the surface of the nanofiber. The constant discharge of cross-linked nanofiber could be as a result of surface diffusion from the medium at the incipient stage as well as the fact that part of the drug remaining was trapped within the pores of the used nanofiber [65]. In addition, after cross-linking, the W_S_CHT/PVA’s linear chain changes to form network chains that have reduced swelling ratio and this significantly decreases the drug’s diffusion from the fiber. Drug release increases when time is increased and this reduces the initial burst release of the drug. The SEM images of drug (hesperetin) loaded samples are shown in Figure 10.

### 3.12. Adsorption Properties

**Figure 11:**
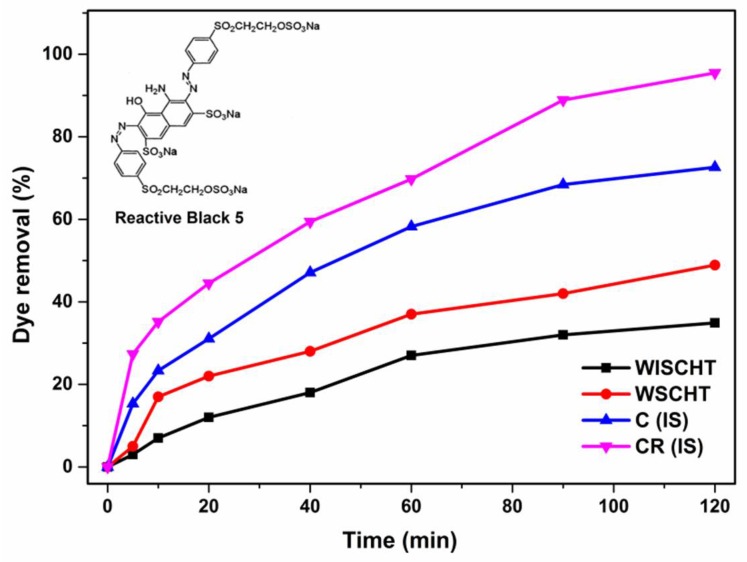
The effect of adsorption time on dye removal by prepared nanofibers and powders.

CHT has various number of free amino and hydroxyl groups and thus is useful for many applications for example, effective removal of organic pollutants and toxic heavy metal ions from waste water [66,67]. But, the disadvantage of pH-dependence restricts its absorbency application due to the zero potential of free amino groups lying within pH (6.5–6.7) that is not allowed to protonate in alkaline solutions [68]. To overcome this drawback, in the present research we prepared water soluble chitosan (W_S_CHT) by hydrogen peroxide with the increased protonation value (7.2) which is better than water soluble chitosan (W_IS_CHT) (6.5). Then W_S_CHT was blended with PVA to make nanofiber and was further cross-linked. The dye adsorption ability of W_IS_CHT, W_S_CHT, the (ideal) nanofiber sample C (IS) and the cross-linked sample CR (IS) are shown in Figure 11.

The experimental data demonstrates the excellent adsorption ability of C(IS) and CR(IS) over those of W_IS_CHT and W_S_CHT. There was rapid adsorption within the first five minutes and then the removal rate decreased until it reached equilibrium. Rapid adsorption within the first five minutes happens due to the various vacant adsorption sites on the nanofibers’ surface [69]. From Figure 11, it can be seen that WISCHT exhibited more RB5 removal percentage than WSCHT at 5 min, 30%. There is 70% of RB5 removal in the (ideal) nanofiber sample. The RB5 removal efficiency can reach 95% in the cross-linked (ideal) sample within 120 min, due to the increased number of interaction bonds within the polymer bulk. More active sites on the fiber surfaces are generated when dye is removal, explained by the increased protonation of W_S_CHT. These protonated amino groups are responsible for creating an ideal environment for high adsorption i.e., the repulsion between the positively charged polymeric chains accelerated the colored liquid transference inside the nanofiber, which attract negative charged dye molecules, this is in agreement of similar findings [70].

## 4. Conclusions

An effective green reduction method of fabrication water soluble chitosan/polyvinyl alcohol (W_S_CHT/PVA) nanofiber at different mass ratios, has been established. FTIR, SEM, XRD, DSC and TGA analyses were used to determine nanofiber structure morphology and properties. The 20/80 blend ratio demonstrates optimum uniform morphology over other ratios. Contact angle measurement revealed the hydrophilicity behaviour of the nanofiber membranes. The cross-linked W_S_CHT/PVA nanofiber showed sustained drug release of hesperetin up to 12 h, and a higher adsorption capacity in RB5 dye measurements. Our data implies that fabricated nanofibers could be employed in many applications to mitigate chemicals that are toxic to the environment, rendering this study as a signpost in future developments of green nanotechnology.

## Figures and Tables

**Table 1:** Properties of solutions.

Sample Code	W_S_CHT:PVA Mass Ratio	Viscosity (cP)	Conductivity (mS/cm)	Surface Tension (mN/m)
a	10:90	3.26	1.26	41.89
b	15:85	3.02	1.32	42.66
c	20:80	2.89	1.53	43.57
d	25:75	2.63	1.62	45.77
e	30:70	2.42	1.88	46.56

**Table 2:** Diameter of prepared nanofibers.

Sample Code	W_S_CHT:PVA Mass Ratio	Diameter (nm)
a	10:90	284.5
b	15:85	179.6
c	20:80	142.4
d	25:75	121.2
e	30:70	99.93
f	20:80	130.6
